# Crystal structure of [bis­(2,6-diiso­propyl­phen­yl) phosphato-κ*O*]tris­(methanol-κ*O*)lithium methanol monosolvate

**DOI:** 10.1107/S2056989015005563

**Published:** 2015-04-02

**Authors:** Mikhail E. Minyaev, Ilya E. Nifant’ev, Alexander N. Tavtorkin, Sof’ya A. Korchagina, Shadana Sh. Zeynalova

**Affiliations:** a A.V. Topchiev Institute of Petrochemical Synthesis, Russian Academy of Sciences, Leninsky prospect 29, 119991 Moscow, Russian Federation; b Moscow City Pedagogical University, 2nd Selskokhozyaistvenny proezd 4, 129226, Moscow, Russian Federation

**Keywords:** crystal structure, alkali metal phosphate diester, diaryl phosphate, hydrogen bonding

## Abstract

Crystals of the title compound, [Li{OOP(O-2,6-^i^Pr_2_C_6_H_3_)_2_}(CH_3_OH)_3_]·CH_3_OH or [Li(C_24_H_34_O_4_P)(CH_3_OH)_3_]·CH_3_OH, have been formed in the reaction between HOOP(O-2,6-^i^Pr_2_C_6_H_3_)_2_ and LiOH in methanol. The title compound is of inter­est as it represents the first reported crystal structure of the family of lithium phosphate diesters. The {Li(CH_3_OH)_3_[O_2_P(O-^i^Pr_2_C_6_H_3_)_2_]} unit displays the Li atom in a slightly distorted tetra­hedral coordination environment and exhibits one intra­molecular O—H⋯O hydrogen bond between a coordinating methanol mol­ecule and the terminal non-coordinating O atom of the phosphate group. The unit is connected with two non-coordinating methanol mol­ecules through two inter­molecular O—H⋯O hydrogen bonds, and with a neighbouring unit through two other O—H⋯O inter­actions. These inter­molecular hydrogen bonds lead to the formation of infinite chains along [100]. There are no significant inter­actions between the chains.

## Abstract 2

In the first reported crystal structure of the family of lithium phosphate diesters, the Li atom is in a slightly distorted tetra­hedral coordination environment and exhibits one intra­molecular O—H⋯O hydrogen bond between a coordinating methanol mol­ecule and the terminal non-coordinating O atom of the phosphate group. The unit is connected with two non-coordinating methanol mol­ecules through two inter­molecular O—H⋯O hydrogen bonds and with a neighbouring unit through two other O—H⋯O inter­actions.

## Chemical context   

Alkali metal phosphate diesters are of inter­est not only because of their fundamental biological importance (see, for example: Gerus & Lis, 2013 ▸, and references therein), but also because they are direct synthetic precursors of organophos­phate *d*- and *f*-metal complexes, which may find applications in various catalytic reactions. For example, rare-earth tris-(diaryl phosphate) complexes may be successfully used in polymerization reactions of 1,4-dienes (Nifant’ev *et al.*, 2013 ▸, 2014 ▸).
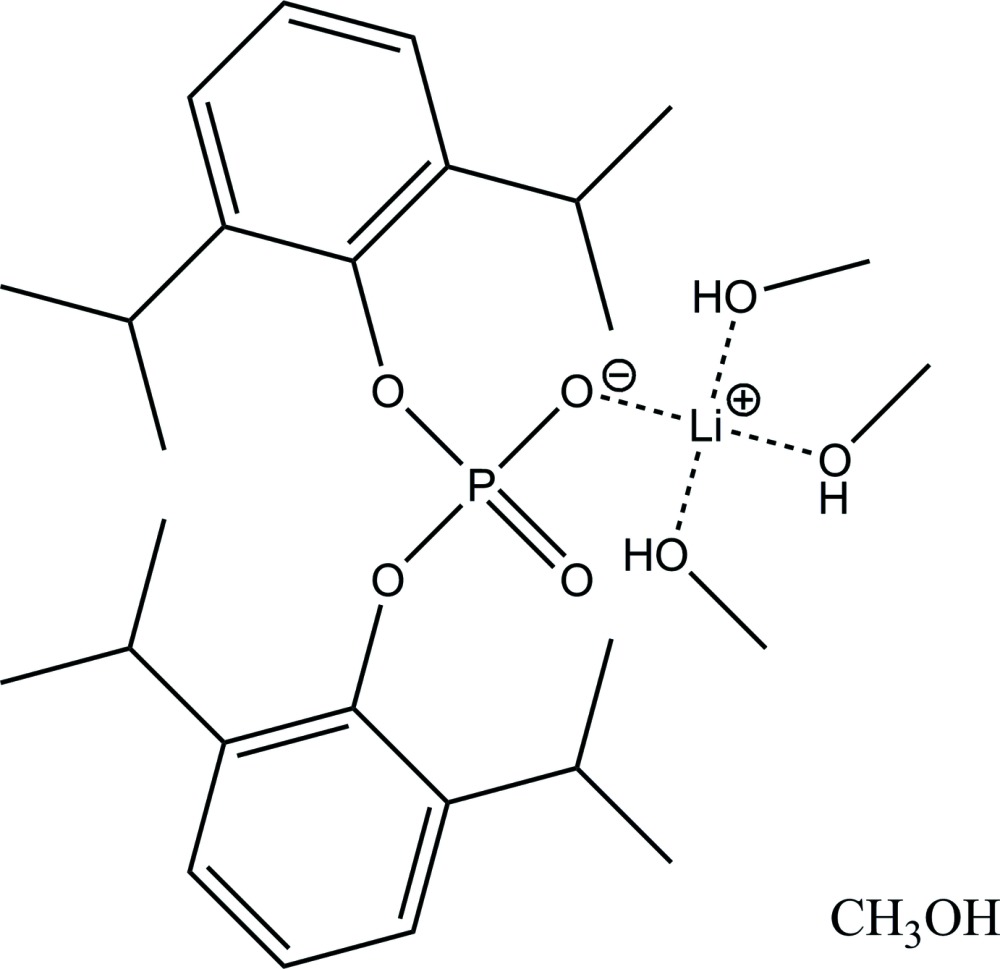


Crystals of the title compound, [Li(CH_3_OH)_3_{OOP(O-2,6-^i^Pr_2_C_6_H_3_)_2_}]·CH_3_OH, have been obtained from the reaction between HOOP(O-2,6-^i^Pr_2_C_6_H_3_)_2_ and LiOH in methanol followed by cooling the reaction mixture. Bis(2,6-diiso­propyl­phen­yl)phospho­ric acid (for its synthesis, see: Blonski *et al.*, 1982 ▸; Kosolapoff *et al.*, 1968 ▸) was prepared from phosphoryl trichloride and 2,6-diiso­propyl­phenol.

## Structural commentary   

**Figure 1:**
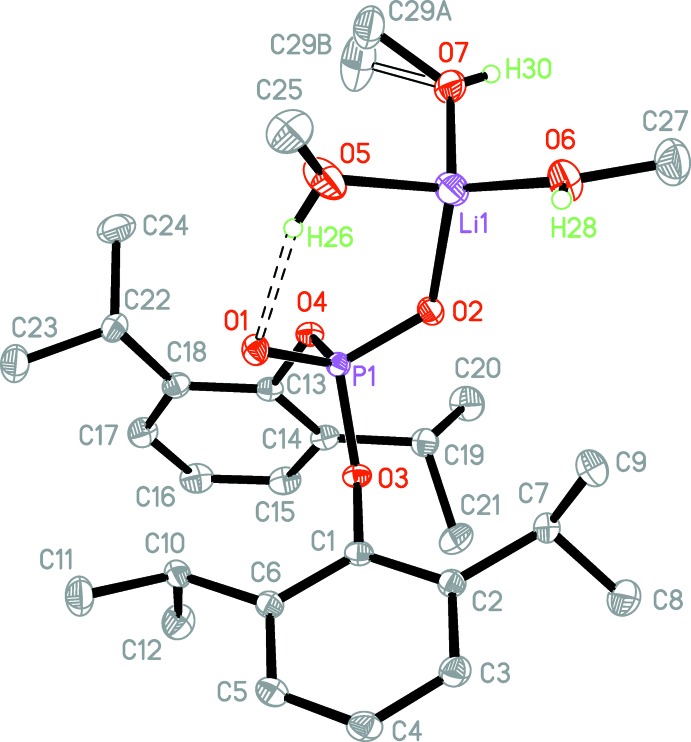
The mol­ecular structure of the {Li(CH_3_OH)_3_[OOP(O-2,6-^i^Pr_2_C_6_H_3_)_2_]} unit. Displacement ellipsoids for non-H atoms are drawn at the 50% probability level. All but hy­droxy hydrogen atoms are omitted for clarity. The intra­molecular hydrogen bond is shown by a dashed line. The minor component of disorder in one of the methanol mol­ecules, C29*B*, is shown with a solid open line.

In the crystal structure of the title solvate, [Li(CH_3_OH)_3_{OOP(O-2,6-^i^Pr_2_C_6_H_3_)_2_}]·CH_3_OH, the {Li(CH_3_OH)_3_[OOP(O-2,6-^i^Pr_2_C_6_H_3_)_2_]} unit contains the Li^+^ cation coordinated by three methanol mol­ecules through the O5, O6 and O7 oxygen atoms (Fig. 1 ▸). One of the coordinating methanol mol­ecules has its methyl group disordered over two positions [occupancy ratio 0.75 (2):0.25 (2)]. The coordination sphere of Li^+^ is completed by the O2 oxygen atom of the diaryl phosphate group, [OOP(O-2,6-^i^Pr_2_C_6_H_3_)_2_]^−^. This configuration is stabilized by an intra­molecular hydrogen bond O5—H26⋯O1 (Fig.1, Table 1 ▸).

**Figure 2:**
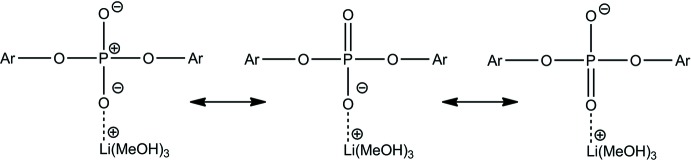
Plausible resonance forms of the [OOP(O-2,6-^i^Pr_2_C_6_H_3_)_2_]^−^ anion.

The phospho­rus and lithium atoms are in approximately tetra­hedral environments with the corresponding bond angles ranging from 99.06 (4)–115.86 (4)° for the phosphate group and 101.32 (9)–118.40 (11) ° for the [LiO_4_] unit. The Li—O bond lengths range from 1.915 (2) Å to 1.945 (2) Å (Table 2 ▸). The P—O bonds can be grouped into two sets. The P1—O1 (P=O) and P1—O2 (P—O—Li) bonds have similar lengths and are ≃ 0.1 Å shorter than the P1—O3 and P1—O4 (P—O—C_*ipso*_) bonds (Table 2 ▸), *i.e.* regular single P—O bonds. Since the O3—C1 and O4—C13 (O—C_*ipso*_) bond lengths also correspond to single bonds, there is no charge redistribution between the PO_4_ core and the two aryl fragments. These observations could best be rationalized by three major resonance forms of the anion (Fig. 2 ▸).

## Supra­molecular features   

All vibrational absorption bands (*e.g.* C—H, C—C, CCH, O—H *etc.*) in the IR spectrum of the solid are fully consistent with the formula with only one exception. Regardless of the O—H absorption bands at 3636, 3576 cm^−1^, the usual methanol C—O absorption bands at 1025–1030 cm^−1^ are missing. A possible explanation is that the methanol mol­ecules are coordinating to lithium and form a hydrogen-bonding network. Consequently, the C—O stretching frequency may be shifted to lower wavenumbers and can be camouflaged by the phosphate absorption band at 912 cm^−1^. This explanation would correspond to the structure data as determined by X-ray diffraction in the current study.

**Figure 3:**
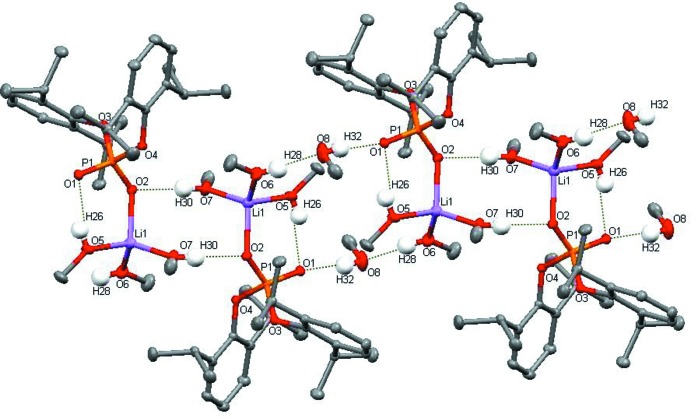
One-dimensional framework of {[Li(CH_3_OH)_3_][OOP(O-2,6-^i^Pr_2_C_6_H_3_)_2_]}(CH_3_OH). All inter­molecular and intra­molecular O—H⋯O hydrogen bonds are shown. All but hy­droxy hydrogen atoms are omitted for clarity. Displacement ellipsoids are drawn at the 50% probability level.

The {[Li(CH_3_OH)_3_][OOP(O-2,6-^i^Pr_2_C_6_H_3_)_2_]} unit is involved in four inter­molecular hydrogen bonds (Table 1 ▸, Fig. 3 ▸). Two symmetry-related O7—H30⋯O2 bonds connect two neighbouring units. O6—H28⋯O8 and O8—H32⋯O1 bonds link one unit and two non-coordinating methanol mol­ecules, which are further connected to another unit. These four inter­molecular hydrogen bonds result in an infinite chain extending along [100], connecting the {[Li(CH_3_OH)_3_][OOP(O-2,6-^i^Pr_2_C_6_H_3_)_2_]} units and non-coordinating methanol mol­ecules. Neighbouring mol­ecules are related by inversion centers. Therefore, the orientations of the cations and anions switch in such a way as to allow the ions of neighbouring mol­ecules in the chains to be involved in additional inter­molecular Coulombic inter­actions (Fig. 3 ▸).

**Figure 4:**
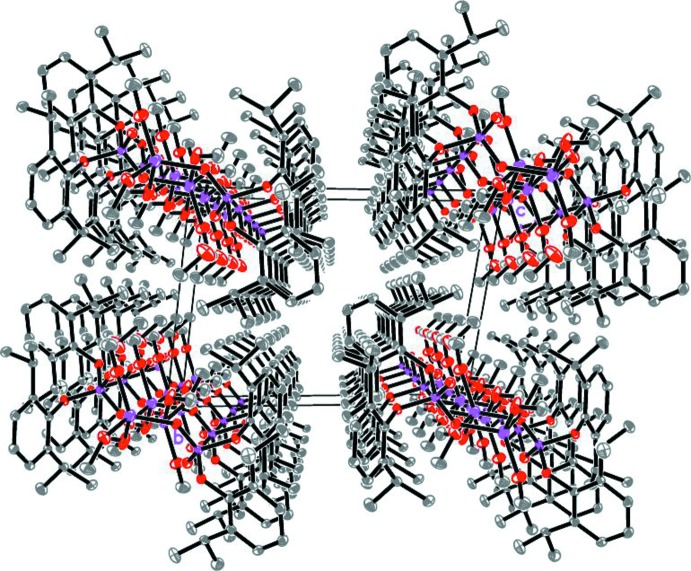
Packing diagram parallel to (100). All H atoms are omitted and hydrogen bonds are not shown. Infinite chains of [{Li(CH_3_OH)_3_[OOP(O-2,6-^i^Pr_2_C_6_H_3_)_2_]}_2_(CH_3_OH)_2_]_*n*_ extend along [100].

The packing of the title compound is shown in Fig. 4 ▸. No significant hydrogen-bonding inter­actions are found between neighbouring chains. However, some short intra­chain contacts between methyl groups are present, probably due to crystal-packing effects.

## Database survey   

According to the Cambridge Structural Database (CSD version 5.35 with updates, Groom & Allen, 2014 ▸), the number of (*R*O)_2_PO_2_ *M*(solv)_x_ structures (*M* is an alkali metal, solv is a solvent mol­ecule) is rather small. Structures containing additional transition metal atoms have been excluded from the search.

For related structures of potassium or sodium phosphate diesters, see: Kumara Swamy *et al.* (2001 ▸), CSD refcode ADAKUL; Gerus & Lis (2013 ▸), AGACIW; Kommana & Swamy (2003 ▸), BEDSOT; Hilken *et al.* (2014 ▸), NIZFEJ; Kumara Swamy & Kumaraswamy (2001 ▸), TIJCUK; Lugmair & Tilley (1998 ▸), VADMES; Ślepokura (2008 ▸), VIVRAU, VIVREY, VIVRIC. A mixed potassium and calcium phosphate diester has been described by Ślepokura (2008 ▸), VIVRUO. All ten found crystal structures are sodium or potassium salts. No lithium compound phosphate diesters has been structurally characterized up to date. Therefore, crystal structures of alkali metal dialkyl and diaryl phosphates remain virtually unexplored.

## Synthesis and crystallization   

**Synthesis of bis­(2,6-diiso­propyl­phen­yl) phospho­ric acid**. Phosphoryl trichloride (12.6 ml, 21.0 g, 137 mmol, *d* = 1.67 g/ml) was added to a stirred solution of 2,6-diiso­propyl­phenol (52.60 g, 295 mmol) in benzene (60 ml). Et_3_N (44.0 ml, 32.0 g, 317 mmol, *d* = 0.728 g/ml, distilled over NaOH prior to use) was carefully added in small parts to the reaction mixture, while it was stirred vigorously. The reaction mixture consisted of a pale-yellow solution and an off-white precipitate of tri­ethyl­amine hydro­chloride. The mixture was heated under reflux for 2 days with occasional stirring. Then, water was added, and after stirring for 1 h, the organic and water layers were separated. The organic phase was evaporated under reduced pressure to produce a yellow oil. A mixture of acetone (85 ml) and water (25 ml, 1.39 mol) was added to the residue. The reaction mixture was then heated under reflux for five hours without stirring. All solvent was evaporated under reduced pressure from the mixture. The resulting precipitate was recrystallized from petroleum ether (70/100, ≃ 250 ml), filtered off, washed with cold (273 K) hexane and dried under dynamic vacuum. The yield of white crystals was 40.89 g (97.70 mmol, 71.3%). Melting point 432–433 K. ^1^H NMR (400 MHz, CDCl_3_): δ = 1.03 [24H, *d*, –CH(C***H_3_***)_2_], 3.34 [4H, septet, –C***H***(CH_3_)_2_], 7.02–7.14 (6H, *m*, ***H_Ar­yl_***), 11.08 [1H, *br.s*, P(O)O***H***]; ^31^P NMR (162 MHz, CDCl_3_): δ = −10.19.

**Synthesis and crystallization of tris­(methanol)-lithium bis­(2,6-diiso­propyl­phen­yl) phosphate methanol solvate.** Bis(2,6-diiso­propyl­phen­yl) phospho­ric acid (15.07 g, 36.0 mmol) was dissolved in methanol (50 ml). Lithium hydroxide (0.86 g, 36 mmol) was added in small parts to the mixture until pH = 7–8. The reaction mixture was filtered, and the resulting solution was placed into a freezer (258 K) for 3 days. The grown crystals were filtered off, washed with cold methanol (≃ 273 K). Several colorless needles were selected for X-ray structure determination analysis. The remaining crystals were dried under dynamic vacuum. Yield 7.72 g (14.0 mmol, 39%). ^1^H NMR (400 MHz, CDCl_3_): δ = 1.10 [24H, *d*, –CH(C***H_3_***)_2_, ^3^ *J*_HH_ = 6.85 Hz], 2.63 (4H, *br.s*, CH_3_O***H***), 3.25 (12H, *s*, C***H_3_***OH), 3.60 [4H, septet, –C***H***(CH_3_)_2_, ^3^ *J*_HH_ = 6.85 Hz], 7.03–7.09 (6H, *m*, ***H_Ar­yl_***). ^31^P NMR (162, MHz, CDCl_3_): δ = −10.23.

## Refinement   

Crystal data, data collection and structure refinement details are summarized in Table 3 ▸. The positions of hy­droxy hydrogen atoms were found from a difference map. These atoms were refined with individual isotropic displacement parameters. All other hydrogen atoms were also found from the difference map but positioned geometrically (C—H distance = 0.95 Å for aromatic, 0.98 Å for methyl, 1.00 Å for –C***H***Me_2_ hydrogen atoms) and refined as riding atoms with relative isotropic displacement parameters [*U*_iso_(H) = 1.2*U*_eq_(C) for aromatic and –C***H***Me_2_, 1.5*U*_eq_(C) for methyl hydrogen atoms]. One of the coordinating methanol mol­ecules showed disorder of its methyl group, with restrained occupancies of 0.75 (2):0.25 (2) for atoms C29*A* and C29*B*. A rotating group model was applied for all methyl groups. Reflection 0 0 1 was obstructed by the beam stop and was omitted from refinement.

## Supplementary Material

Crystal structure: contains datablock(s) I. DOI: 10.1107/S2056989015005563/wm5137sup1.cif

Structure factors: contains datablock(s) I. DOI: 10.1107/S2056989015005563/wm5137Isup2.hkl

Supporting information file. DOI: 10.1107/S2056989015005563/wm5137Isup3.cdx

CCDC reference: 1054771

Additional supporting information:  crystallographic information; 3D view; checkCIF report

## Figures and Tables

**Table 1:** Hydrogen-bond geometry (, )

*D*H*A*	*D*H	H*A*	*D* *A*	*D*H*A*
O5H26O1	0.79(2)	2.00(2)	2.7482(11)	158.2(19)
O6H28O8^i^	0.85(2)	1.86(2)	2.7013(14)	174(2)
O7H30O2^ii^	0.83(2)	1.89(2)	2.7152(11)	170.7(19)
O8H32O1	0.82(2)	1.88(2)	2.6929(12)	171.8(19)

Symmetry codes: (i) 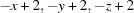; (ii) 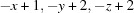.

**Table 2:** Selected bond lengths ()

P1O1	1.4934(7)	Li1O5	1.932(2)
P1O2	1.4965(7)	Li1O6	1.915(2)
P1O3	1.5993(7)	Li1O7	1.931(2)
P1O4	1.6003(7)	O3C1	1.4035(11)
Li1O2	1.945(2)	O4C13	1.4040(11)

**Table 3:** Experimental details

Crystal data	
Chemical formula	[Li(C_24_H_34_O_4_P)(CH_4_O)_3_]CH_4_O
*M*_r_	552.59
Crystal system, space group	Triclinic, *P* 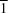
Temperature (K)	123
*a*, *b*, *c* ()	11.1853(6), 11.5046(6), 14.5237(7)
, , ()	90.855(2), 102.859(2), 118.683(1)
*V* (^3^)	1582.21(14)
*Z*	2
Radiation type	Mo *K*
(mm^1^)	0.13
Crystal size (mm)	0.60 0.20 0.10

Data collection	
Diffractometer	Bruker APEXII CCD area-detector
Absorption correction	Multi-scan (*SADABS*; Bruker, 2003 ▸)
*T*_min_, *T*_max_	0.872, 0.986
No. of measured, independent and observed [*I* > 2(*I*)] reflections	22920, 10353, 8544
*R*_int_	0.021
(sin /)_max_ (^1^)	0.732

Refinement	
*R*[*F* ^2^ > 2(*F* ^2^)], *wR*(*F* ^2^), *S*	0.040, 0.112, 1.02
No. of reflections	10353
No. of parameters	382
H-atom treatment	H atoms treated by a mixture of independent and constrained refinement
_max_, _min_ (e ^3^)	0.77, 0.53

Computer programs: *APEX2* and *SAINT* (Bruker, 2007 ▸), *SHELXS2013* and *SHELXTL* (Sheldrick, 2008 ▸), *SHELXL2014* (Sheldrick, 2015 ▸), *Mercury* (Macrae *et al.*, 2006 ▸) and *publCIF* (Westrip, 2010 ▸).

## supplementary crystallographic information

### Crystal data

**Table:**

[Li(C_24_H_34_O_4_P)(CH_4_O)_3_]·CH_4_O	*Z* = 2
*M**_r_* = 552.59	*F*(000) = 600
Triclinic, *P*1	*D*_x_ = 1.160 Mg m^−^^3^
*a* = 11.1853 (6) Å	Mo *K*α radiation, λ = 0.710730 (9) Å
*b* = 11.5046 (6) Å	Cell parameters from 9855 reflections
*c* = 14.5237 (7) Å	θ = 2.3–31.3°
α = 90.855 (2)°	µ = 0.13 mm^−^^1^
β = 102.859 (2)°	*T* = 123 K
γ = 118.683 (1)°	Needle, colorless
*V* = 1582.21 (14) Å^3^	0.60 × 0.20 × 0.10 mm

### Data collection

**Table:**

Bruker APEXII CCD area-detector diffractometer	10353 independent reflections
Radiation source: fine-focus sealed tube	8544 reflections with *I* > 2σ(*I*)
Graphite monochromator	*R*_int_ = 0.021
ω scans	θ_max_ = 31.3°, θ_min_ = 2.0°
Absorption correction: multi-scan (*SADABS*; Bruker, 2003)	*h* = −16→16
*T*_min_ = 0.872, *T*_max_ = 0.986	*k* = −16→16
22920 measured reflections	*l* = −21→21

### Refinement

**Table:**

Refinement on *F*^2^	Primary atom site location: structure-invariant direct methods
Least-squares matrix: full	Secondary atom site location: difference Fourier map
*R*[*F*^2^ > 2σ(*F*^2^)] = 0.040	Hydrogen site location: mixed
*wR*(*F*^2^) = 0.112	H atoms treated by a mixture of independent and constrained refinement
*S* = 1.02	*w* = 1/[σ^2^(*F*_o_^2^) + (0.0628*P*)^2^ + 0.329*P*] where *P* = (*F*_o_^2^ + 2*F*_c_^2^)/3
10353 reflections	(Δ/σ)_max_ = 0.001
382 parameters	Δρ_max_ = 0.77 e Å^−^^3^
0 restraints	Δρ_min_ = −0.53 e Å^−^^3^

### Special details

**Table:**

Geometry. All e.s.d.'s (except the e.s.d. in the dihedral angle between two l.s. planes) are estimated using the full covariance matrix. The cell e.s.d.'s are taken into account individually in the estimation of e.s.d.'s in distances, angles and torsion angles; correlations between e.s.d.'s in cell parameters are only used when they are defined by crystal symmetry. An approximate (isotropic) treatment of cell e.s.d.'s is used for estimating e.s.d.'s involving l.s. planes.

### Fractional atomic coordinates and isotropic or equivalent isotropic displacement parameters (Å^2^)

**Table:**

	*x*	*y*	*z*	*U*_iso_*/*U*_eq_	Occ. (<1)
P1	0.62300 (2)	0.88864 (2)	0.82666 (2)	0.01132 (6)	
Li1	0.7224 (2)	1.0298 (2)	1.03455 (14)	0.0216 (4)	
O1	0.71418 (7)	0.82744 (7)	0.85725 (5)	0.01552 (14)	
O2	0.64407 (7)	0.99757 (7)	0.89696 (5)	0.01510 (13)	
O3	0.63126 (7)	0.93432 (7)	0.72374 (5)	0.01310 (13)	
O4	0.45827 (7)	0.78087 (7)	0.80228 (5)	0.01346 (13)	
C1	0.75457 (9)	0.99858 (9)	0.69226 (6)	0.01272 (16)	
C2	0.82677 (10)	1.13862 (9)	0.70219 (7)	0.01421 (17)	
C3	0.94480 (11)	1.19980 (10)	0.66505 (7)	0.01713 (18)	
H3A	0.9972	1.2947	0.6711	0.021*	
C4	0.98609 (11)	1.12365 (10)	0.61952 (7)	0.01835 (19)	
H4A	1.0670	1.1667	0.5954	0.022*	
C5	0.90964 (11)	0.98439 (10)	0.60897 (7)	0.01706 (18)	
H5A	0.9383	0.9336	0.5767	0.020*	
C6	0.79173 (10)	0.91821 (9)	0.64503 (7)	0.01379 (17)	
C7	0.78093 (11)	1.22290 (10)	0.75072 (7)	0.01677 (18)	
H7A	0.6876	1.1609	0.7628	0.020*	
C8	0.76209 (14)	1.32155 (12)	0.68757 (9)	0.0267 (2)	
H8A	0.6950	1.2724	0.6260	0.040*	
H8B	0.8533	1.3862	0.6772	0.040*	
H8C	0.7259	1.3691	0.7191	0.040*	
C9	0.88650 (13)	1.29710 (11)	0.84741 (8)	0.0234 (2)	
H9A	0.8916	1.2320	0.8885	0.035*	
H9B	0.8554	1.3507	0.8777	0.035*	
H9C	0.9800	1.3561	0.8377	0.035*	
C10	0.70174 (10)	0.76631 (10)	0.62834 (7)	0.01561 (17)	
H10A	0.6566	0.7390	0.6823	0.019*	
C11	0.78739 (13)	0.69543 (11)	0.62659 (9)	0.0243 (2)	
H11A	0.8629	0.7261	0.6858	0.036*	
H11B	0.8287	0.7163	0.5721	0.036*	
H11C	0.7253	0.5985	0.6207	0.036*	
C12	0.58316 (12)	0.72179 (11)	0.53609 (8)	0.0228 (2)	
H12A	0.5282	0.7665	0.5397	0.034*	
H12B	0.5212	0.6246	0.5281	0.034*	
H12C	0.6242	0.7459	0.4816	0.034*	
C13	0.37172 (10)	0.70309 (9)	0.71375 (7)	0.01358 (16)	
C14	0.30293 (10)	0.75513 (10)	0.64912 (7)	0.01566 (17)	
C15	0.20879 (11)	0.67082 (11)	0.56457 (7)	0.01988 (19)	
H15A	0.1599	0.7025	0.5193	0.024*	
C16	0.18558 (11)	0.54178 (11)	0.54579 (8)	0.0221 (2)	
H16A	0.1214	0.4862	0.4881	0.027*	
C17	0.25624 (11)	0.49414 (10)	0.61134 (8)	0.02018 (19)	
H17A	0.2401	0.4060	0.5977	0.024*	
C18	0.35065 (10)	0.57348 (10)	0.69699 (7)	0.01573 (17)	
C19	0.32711 (11)	0.89620 (10)	0.66694 (7)	0.01742 (18)	
H19A	0.3980	0.9406	0.7299	0.021*	
C20	0.19082 (13)	0.89471 (14)	0.67174 (9)	0.0277 (2)	
H20A	0.1560	0.8457	0.7232	0.042*	
H20B	0.1194	0.8506	0.6109	0.042*	
H20C	0.2100	0.9868	0.6842	0.042*	
C21	0.38705 (12)	0.97881 (12)	0.58994 (8)	0.0239 (2)	
H21A	0.4731	0.9779	0.5866	0.036*	
H21B	0.4093	1.0713	0.6061	0.036*	
H21C	0.3170	0.9400	0.5280	0.036*	
C22	0.42568 (11)	0.52122 (10)	0.77067 (7)	0.01739 (18)	
H22A	0.5211	0.5984	0.8025	0.021*	
C23	0.44768 (15)	0.41384 (12)	0.72666 (9)	0.0283 (2)	
H23A	0.4917	0.4461	0.6742	0.042*	
H23B	0.3563	0.3322	0.7022	0.042*	
H23C	0.5092	0.3944	0.7754	0.042*	
C24	0.34634 (14)	0.46943 (14)	0.84766 (9)	0.0294 (3)	
H24A	0.3386	0.5414	0.8779	0.044*	
H24B	0.3981	0.4396	0.8959	0.044*	
H24C	0.2516	0.3941	0.8185	0.044*	
O5	0.81065 (10)	0.91990 (11)	1.04939 (6)	0.0312 (2)	
C25	0.92769 (13)	0.92434 (13)	1.11483 (9)	0.0284 (2)	
H25A	0.9020	0.8346	1.1315	0.043*	
H25B	0.9552	0.9860	1.1726	0.043*	
H25C	1.0070	0.9554	1.0857	0.043*	
O6	0.86571 (9)	1.20747 (8)	1.09305 (6)	0.02504 (17)	
C27	0.84869 (16)	1.31713 (13)	1.11945 (11)	0.0339 (3)	
H27A	0.8725	1.3800	1.0729	0.051*	
H27B	0.9113	1.3628	1.1829	0.051*	
H27C	0.7505	1.2842	1.1207	0.051*	
O7	0.56431 (9)	0.94990 (8)	1.09033 (6)	0.02329 (17)	
C29A	0.5209 (6)	0.8252 (4)	1.1245 (4)	0.0311 (8)	0.75 (2)
H29A	0.5812	0.7894	1.1136	0.047*	0.75 (2)
H29B	0.4225	0.7624	1.0905	0.047*	0.75 (2)
H29C	0.5290	0.8372	1.1929	0.047*	0.75 (2)
C29B	0.4900 (14)	0.8055 (11)	1.084 (2)	0.044 (3)	0.25 (2)
H29D	0.4135	0.7671	1.0245	0.066*	0.25 (2)
H29E	0.4504	0.7794	1.1384	0.066*	0.25 (2)
H29F	0.5558	0.7723	1.0829	0.066*	0.25 (2)
O8	0.85286 (11)	0.68825 (11)	0.87815 (8)	0.0382 (2)	
C31	0.78917 (14)	0.59808 (13)	0.93888 (10)	0.0308 (3)	
H31A	0.7034	0.5192	0.9011	0.046*	
H31B	0.7646	0.6413	0.9843	0.046*	
H31C	0.8550	0.5706	0.9738	0.046*	
H26	0.799 (2)	0.8875 (19)	0.9979 (15)	0.045 (5)*	
H28	0.953 (2)	1.236 (2)	1.0986 (14)	0.051 (5)*	
H30	0.507 (2)	0.9752 (19)	1.0954 (14)	0.049 (5)*	
H32	0.814 (2)	0.7331 (19)	0.8674 (14)	0.047 (5)*	

### Atomic displacement parameters (Å^2^)

**Table:**

	*U*^11^	*U*^22^	*U*^33^	*U*^12^	*U*^13^	*U*^23^
P1	0.01157 (11)	0.01212 (11)	0.01118 (11)	0.00666 (8)	0.00276 (8)	0.00173 (8)
Li1	0.0244 (9)	0.0239 (9)	0.0188 (8)	0.0142 (8)	0.0046 (7)	0.0014 (7)
O1	0.0157 (3)	0.0174 (3)	0.0161 (3)	0.0110 (3)	0.0025 (3)	0.0020 (3)
O2	0.0175 (3)	0.0154 (3)	0.0139 (3)	0.0094 (3)	0.0039 (2)	0.0008 (2)
O3	0.0117 (3)	0.0157 (3)	0.0126 (3)	0.0066 (2)	0.0048 (2)	0.0041 (2)
O4	0.0119 (3)	0.0149 (3)	0.0119 (3)	0.0055 (2)	0.0028 (2)	0.0017 (2)
C1	0.0109 (4)	0.0150 (4)	0.0121 (4)	0.0060 (3)	0.0034 (3)	0.0024 (3)
C2	0.0147 (4)	0.0143 (4)	0.0132 (4)	0.0070 (3)	0.0033 (3)	0.0018 (3)
C3	0.0159 (4)	0.0147 (4)	0.0177 (4)	0.0052 (3)	0.0048 (3)	0.0021 (3)
C4	0.0145 (4)	0.0194 (5)	0.0195 (5)	0.0059 (4)	0.0075 (3)	0.0031 (4)
C5	0.0160 (4)	0.0196 (4)	0.0168 (4)	0.0091 (4)	0.0060 (3)	0.0016 (3)
C6	0.0135 (4)	0.0149 (4)	0.0126 (4)	0.0070 (3)	0.0028 (3)	0.0013 (3)
C7	0.0202 (4)	0.0146 (4)	0.0178 (4)	0.0098 (4)	0.0064 (4)	0.0027 (3)
C8	0.0362 (6)	0.0272 (6)	0.0286 (6)	0.0231 (5)	0.0124 (5)	0.0112 (4)
C9	0.0284 (5)	0.0191 (5)	0.0205 (5)	0.0106 (4)	0.0056 (4)	−0.0021 (4)
C10	0.0174 (4)	0.0139 (4)	0.0160 (4)	0.0077 (4)	0.0056 (3)	0.0008 (3)
C11	0.0265 (5)	0.0194 (5)	0.0326 (6)	0.0147 (4)	0.0100 (4)	0.0039 (4)
C12	0.0221 (5)	0.0180 (5)	0.0215 (5)	0.0068 (4)	0.0008 (4)	−0.0004 (4)
C13	0.0107 (4)	0.0145 (4)	0.0132 (4)	0.0044 (3)	0.0034 (3)	0.0021 (3)
C14	0.0131 (4)	0.0188 (4)	0.0152 (4)	0.0079 (4)	0.0040 (3)	0.0045 (3)
C15	0.0155 (4)	0.0248 (5)	0.0167 (4)	0.0092 (4)	0.0012 (3)	0.0041 (4)
C16	0.0174 (5)	0.0225 (5)	0.0180 (5)	0.0051 (4)	0.0005 (4)	0.0000 (4)
C17	0.0183 (4)	0.0157 (4)	0.0198 (5)	0.0043 (4)	0.0025 (4)	0.0003 (4)
C18	0.0139 (4)	0.0148 (4)	0.0163 (4)	0.0053 (3)	0.0044 (3)	0.0035 (3)
C19	0.0170 (4)	0.0208 (5)	0.0179 (4)	0.0123 (4)	0.0039 (3)	0.0044 (4)
C20	0.0249 (5)	0.0372 (6)	0.0314 (6)	0.0226 (5)	0.0089 (5)	0.0077 (5)
C21	0.0248 (5)	0.0250 (5)	0.0236 (5)	0.0137 (4)	0.0062 (4)	0.0105 (4)
C22	0.0187 (4)	0.0143 (4)	0.0184 (4)	0.0078 (4)	0.0040 (4)	0.0037 (3)
C23	0.0388 (7)	0.0247 (5)	0.0270 (6)	0.0213 (5)	0.0058 (5)	0.0031 (4)
C24	0.0357 (6)	0.0361 (6)	0.0252 (6)	0.0215 (6)	0.0146 (5)	0.0156 (5)
O5	0.0379 (5)	0.0508 (6)	0.0161 (4)	0.0356 (5)	−0.0045 (3)	−0.0052 (4)
C25	0.0228 (5)	0.0342 (6)	0.0244 (5)	0.0151 (5)	−0.0029 (4)	0.0048 (5)
O6	0.0241 (4)	0.0222 (4)	0.0292 (4)	0.0115 (3)	0.0080 (3)	−0.0014 (3)
C27	0.0407 (7)	0.0261 (6)	0.0443 (8)	0.0197 (6)	0.0210 (6)	0.0057 (5)
O7	0.0300 (4)	0.0268 (4)	0.0267 (4)	0.0213 (4)	0.0144 (3)	0.0105 (3)
C29A	0.0370 (16)	0.0231 (11)	0.0440 (17)	0.0190 (11)	0.0200 (14)	0.0109 (11)
C29B	0.034 (4)	0.026 (4)	0.080 (11)	0.017 (3)	0.025 (5)	0.013 (5)
O8	0.0365 (5)	0.0451 (6)	0.0568 (7)	0.0329 (5)	0.0243 (5)	0.0282 (5)
C31	0.0285 (6)	0.0269 (6)	0.0361 (7)	0.0144 (5)	0.0046 (5)	0.0098 (5)

### Geometric parameters (Å, º)

**Table:**

P1—O1	1.4934 (7)	C16—H16A	0.9500
P1—O2	1.4965 (7)	C17—C18	1.3971 (14)
P1—O3	1.5993 (7)	C17—H17A	0.9500
P1—O4	1.6003 (7)	C18—C22	1.5214 (14)
Li1—O2	1.945 (2)	C19—C20	1.5335 (15)
Li1—O5	1.932 (2)	C19—C21	1.5352 (15)
Li1—O6	1.915 (2)	C19—H19A	1.0000
Li1—O7	1.931 (2)	C20—H20A	0.9800
O3—C1	1.4035 (11)	C20—H20B	0.9800
O4—C13	1.4040 (11)	C20—H20C	0.9800
C1—C2	1.4003 (13)	C21—H21A	0.9800
C1—C6	1.4053 (13)	C21—H21B	0.9800
C2—C3	1.4019 (13)	C21—H21C	0.9800
C2—C7	1.5224 (13)	C22—C23	1.5276 (15)
C3—C4	1.3875 (14)	C22—C24	1.5320 (15)
C3—H3A	0.9500	C22—H22A	1.0000
C4—C5	1.3930 (14)	C23—H23A	0.9800
C4—H4A	0.9500	C23—H23B	0.9800
C5—C6	1.3960 (13)	C23—H23C	0.9800
C5—H5A	0.9500	C24—H24A	0.9800
C6—C10	1.5219 (13)	C24—H24B	0.9800
C7—C8	1.5325 (15)	C24—H24C	0.9800
C7—C9	1.5342 (15)	O5—C25	1.4144 (14)
C7—H7A	1.0000	O5—H26	0.79 (2)
C8—H8A	0.9800	C25—H25A	0.9800
C8—H8B	0.9800	C25—H25B	0.9800
C8—H8C	0.9800	C25—H25C	0.9800
C9—H9A	0.9800	O6—C27	1.4225 (14)
C9—H9B	0.9800	O6—H28	0.85 (2)
C9—H9C	0.9800	C27—H27A	0.9800
C10—C11	1.5309 (14)	C27—H27B	0.9800
C10—C12	1.5339 (15)	C27—H27C	0.9800
C10—H10A	1.0000	O7—C29A	1.415 (3)
C11—H11A	0.9800	O7—C29B	1.447 (11)
C11—H11B	0.9800	O7—H30	0.83 (2)
C11—H11C	0.9800	C29A—H29A	0.9800
C12—H12A	0.9800	C29A—H29B	0.9800
C12—H12B	0.9800	C29A—H29C	0.9800
C12—H12C	0.9800	C29B—H29D	0.9800
C13—C18	1.4016 (14)	C29B—H29E	0.9800
C13—C14	1.4032 (13)	C29B—H29F	0.9800
C14—C15	1.4021 (14)	O8—C31	1.3996 (16)
C14—C19	1.5196 (14)	O8—H32	0.82 (2)
C15—C16	1.3901 (16)	C31—H31A	0.9800
C15—H15A	0.9500	C31—H31B	0.9800
C16—C17	1.3885 (15)	C31—H31C	0.9800
			
O1—P1—O2	115.86 (4)	C18—C17—H17A	119.4
O1—P1—O3	110.98 (4)	C17—C18—C13	117.37 (9)
O2—P1—O3	111.66 (4)	C17—C18—C22	121.75 (9)
O1—P1—O4	112.44 (4)	C13—C18—C22	120.87 (9)
O2—P1—O4	105.46 (4)	C14—C19—C20	111.38 (9)
O3—P1—O4	99.06 (4)	C14—C19—C21	111.12 (9)
O6—Li1—O7	114.81 (10)	C20—C19—C21	110.04 (9)
O6—Li1—O5	106.80 (10)	C14—C19—H19A	108.1
O7—Li1—O5	107.49 (10)	C20—C19—H19A	108.1
O6—Li1—O2	118.40 (11)	C21—C19—H19A	108.1
O7—Li1—O2	106.73 (10)	C19—C20—H20A	109.5
O5—Li1—O2	101.32 (9)	C19—C20—H20B	109.5
P1—O2—Li1	128.08 (7)	H20A—C20—H20B	109.5
C1—O3—P1	125.61 (6)	C19—C20—H20C	109.5
C13—O4—P1	126.90 (6)	H20A—C20—H20C	109.5
C2—C1—O3	118.35 (8)	H20B—C20—H20C	109.5
C2—C1—C6	123.54 (9)	C19—C21—H21A	109.5
O3—C1—C6	117.87 (8)	C19—C21—H21B	109.5
C1—C2—C3	117.12 (9)	H21A—C21—H21B	109.5
C1—C2—C7	122.36 (9)	C19—C21—H21C	109.5
C3—C2—C7	120.52 (9)	H21A—C21—H21C	109.5
C4—C3—C2	120.88 (9)	H21B—C21—H21C	109.5
C4—C3—H3A	119.6	C18—C22—C23	113.01 (9)
C2—C3—H3A	119.6	C18—C22—C24	110.50 (9)
C3—C4—C5	120.38 (9)	C23—C22—C24	110.65 (9)
C3—C4—H4A	119.8	C18—C22—H22A	107.5
C5—C4—H4A	119.8	C23—C22—H22A	107.5
C4—C5—C6	121.16 (9)	C24—C22—H22A	107.5
C4—C5—H5A	119.4	C22—C23—H23A	109.5
C6—C5—H5A	119.4	C22—C23—H23B	109.5
C5—C6—C1	116.89 (9)	H23A—C23—H23B	109.5
C5—C6—C10	121.63 (8)	C22—C23—H23C	109.5
C1—C6—C10	121.37 (8)	H23A—C23—H23C	109.5
C2—C7—C8	111.88 (8)	H23B—C23—H23C	109.5
C2—C7—C9	110.51 (8)	C22—C24—H24A	109.5
C8—C7—C9	110.68 (9)	C22—C24—H24B	109.5
C2—C7—H7A	107.9	H24A—C24—H24B	109.5
C8—C7—H7A	107.9	C22—C24—H24C	109.5
C9—C7—H7A	107.9	H24A—C24—H24C	109.5
C7—C8—H8A	109.5	H24B—C24—H24C	109.5
C7—C8—H8B	109.5	C25—O5—Li1	135.51 (10)
H8A—C8—H8B	109.5	C25—O5—H26	111.9 (14)
C7—C8—H8C	109.5	Li1—O5—H26	106.6 (14)
H8A—C8—H8C	109.5	O5—C25—H25A	109.5
H8B—C8—H8C	109.5	O5—C25—H25B	109.5
C7—C9—H9A	109.5	H25A—C25—H25B	109.5
C7—C9—H9B	109.5	O5—C25—H25C	109.5
H9A—C9—H9B	109.5	H25A—C25—H25C	109.5
C7—C9—H9C	109.5	H25B—C25—H25C	109.5
H9A—C9—H9C	109.5	C27—O6—Li1	128.27 (10)
H9B—C9—H9C	109.5	C27—O6—H28	108.3 (14)
C6—C10—C11	113.23 (8)	Li1—O6—H28	122.7 (13)
C6—C10—C12	109.69 (8)	O6—C27—H27A	109.5
C11—C10—C12	110.98 (9)	O6—C27—H27B	109.5
C6—C10—H10A	107.6	H27A—C27—H27B	109.5
C11—C10—H10A	107.6	O6—C27—H27C	109.5
C12—C10—H10A	107.6	H27A—C27—H27C	109.5
C10—C11—H11A	109.5	H27B—C27—H27C	109.5
C10—C11—H11B	109.5	C29A—O7—Li1	122.16 (15)
H11A—C11—H11B	109.5	C29B—O7—Li1	117.0 (7)
C10—C11—H11C	109.5	C29A—O7—H30	108.0 (14)
H11A—C11—H11C	109.5	C29B—O7—H30	106.9 (14)
H11B—C11—H11C	109.5	Li1—O7—H30	129.6 (14)
C10—C12—H12A	109.5	O7—C29A—H29A	109.5
C10—C12—H12B	109.5	O7—C29A—H29B	109.5
H12A—C12—H12B	109.5	H29A—C29A—H29B	109.5
C10—C12—H12C	109.5	O7—C29A—H29C	109.5
H12A—C12—H12C	109.5	H29A—C29A—H29C	109.5
H12B—C12—H12C	109.5	H29B—C29A—H29C	109.5
C18—C13—C14	123.15 (9)	O7—C29B—H29D	109.5
C18—C13—O4	118.19 (8)	O7—C29B—H29E	109.5
C14—C13—O4	118.51 (8)	H29D—C29B—H29E	109.5
C15—C14—C13	117.02 (9)	O7—C29B—H29F	109.5
C15—C14—C19	119.91 (9)	H29D—C29B—H29F	109.5
C13—C14—C19	123.07 (9)	H29E—C29B—H29F	109.5
C16—C15—C14	121.25 (10)	C31—O8—H32	108.1 (14)
C16—C15—H15A	119.4	O8—C31—H31A	109.5
C14—C15—H15A	119.4	O8—C31—H31B	109.5
C17—C16—C15	120.01 (10)	H31A—C31—H31B	109.5
C17—C16—H16A	120.0	O8—C31—H31C	109.5
C15—C16—H16A	120.0	H31A—C31—H31C	109.5
C16—C17—C18	121.20 (10)	H31B—C31—H31C	109.5
C16—C17—H17A	119.4		
			
O1—P1—O2—Li1	−22.98 (11)	C3—C2—C7—C9	−70.95 (12)
O3—P1—O2—Li1	−151.34 (9)	C5—C6—C10—C11	34.55 (13)
O4—P1—O2—Li1	102.06 (10)	C1—C6—C10—C11	−149.58 (9)
O1—P1—O3—C1	−42.03 (8)	C5—C6—C10—C12	−90.02 (11)
O2—P1—O3—C1	88.89 (8)	C1—C6—C10—C12	85.85 (11)
O4—P1—O3—C1	−160.39 (7)	P1—O4—C13—C18	95.20 (10)
Li1—P1—O3—C1	68.81 (10)	P1—O4—C13—C14	−89.13 (10)
O1—P1—O4—C13	−88.42 (8)	C18—C13—C14—C15	0.30 (14)
O2—P1—O4—C13	144.44 (7)	O4—C13—C14—C15	−175.14 (8)
O3—P1—O4—C13	28.84 (8)	C18—C13—C14—C19	−179.32 (9)
Li1—P1—O4—C13	175.22 (8)	O4—C13—C14—C19	5.24 (14)
P1—O3—C1—C2	−94.01 (10)	C13—C14—C15—C16	−0.31 (15)
P1—O3—C1—C6	91.40 (10)	C19—C14—C15—C16	179.32 (10)
O3—C1—C2—C3	−176.33 (8)	C14—C15—C16—C17	0.00 (17)
C6—C1—C2—C3	−2.06 (14)	C15—C16—C17—C18	0.36 (17)
O3—C1—C2—C7	3.54 (13)	C16—C17—C18—C13	−0.38 (15)
C6—C1—C2—C7	177.81 (9)	C16—C17—C18—C22	178.27 (10)
C1—C2—C3—C4	0.79 (14)	C14—C13—C18—C17	0.04 (15)
C7—C2—C3—C4	−179.08 (9)	O4—C13—C18—C17	175.49 (8)
C2—C3—C4—C5	0.71 (16)	C14—C13—C18—C22	−178.61 (9)
C3—C4—C5—C6	−1.07 (16)	O4—C13—C18—C22	−3.16 (13)
C4—C5—C6—C1	−0.11 (14)	C15—C14—C19—C20	62.20 (13)
C4—C5—C6—C10	175.93 (9)	C13—C14—C19—C20	−118.20 (11)
C2—C1—C6—C5	1.72 (14)	C15—C14—C19—C21	−60.88 (12)
O3—C1—C6—C5	176.02 (8)	C13—C14—C19—C21	118.73 (10)
C2—C1—C6—C10	−174.33 (9)	C17—C18—C22—C23	28.37 (14)
O3—C1—C6—C10	−0.04 (13)	C13—C18—C22—C23	−153.04 (10)
C1—C2—C7—C8	−127.00 (10)	C17—C18—C22—C24	−96.23 (12)
C3—C2—C7—C8	52.86 (13)	C13—C18—C22—C24	82.36 (12)
C1—C2—C7—C9	109.19 (11)		

### Hydrogen-bond geometry (Å, º)

**Table:**

*D*—H···*A*	*D*—H	H···*A*	*D*···*A*	*D*—H···*A*
O5—H26···O1	0.79 (2)	2.00 (2)	2.7482 (11)	158.2 (19)
O6—H28···O8^i^	0.85 (2)	1.86 (2)	2.7013 (14)	174 (2)
O7—H30···O2^ii^	0.83 (2)	1.89 (2)	2.7152 (11)	170.7 (19)
O8—H32···O1	0.82 (2)	1.88 (2)	2.6929 (12)	171.8 (19)

Symmetry codes: (i) −*x*+2, −*y*+2, −*z*+2; (ii) −*x*+1, −*y*+2, −*z*+2.
